# Modification of Mechanical Properties of Expansive Soil from North China by Using Rice Husk Ash

**DOI:** 10.3390/ma14112789

**Published:** 2021-05-24

**Authors:** Mazahir M. M. Taha, Cheng-Pei Feng, Sara H. S. Ahmed

**Affiliations:** 1 Civil Engineering Department, Northeast Forestry University, Harbin 150040, China; 2 Civil Engineering Department, Alzaiem Alazhari University, Khartoum 1432, Sudan; 3 Civil Engineering and Architecture, Zhejiang University, Hangzhou 310058, China; sarahassansaad@hotmail.com

**Keywords:** expansive soil, consolidation, coefficient of consolidation, hydraulic conductivity, rice husk ash

## Abstract

The construction of buildings on expansive soils poses considerable risk of damage or collapse due to soil shrinkage or swelling made likely by the remarkable degree compressibility and weak shear resistance of such soils. In this research, rice husk ash (RHA) was added to expansive soil samples in different quantities of 0%, 4%, 8%, 12%, and 16% by weight of soil to determine their effects on the plasticity index, compaction parameters, consolidation performance, and California bearing ratio (CBR)of clay soil. The results show that the use of RHA increases the effective stress and decreases the void ratio and coefficient of consolidation. Adding 16% RHA resulted in the greatest reduction in the hydraulic conductivity, void ratio, and coefficient of consolidation. The void ratio decreased from 0.96 to 0.93, consolidation coefficient decreased from 2.52 to 2.33 cm^2^/s, and hydraulic conductivity decreased from 1.12 to 0.80 cm/s. The addition of RHA improved the soil properties and coefficient of consolidation due to the high density and cohesiveness of RHA. The results of this study can be used to provide a suitable basis for the treatment of expansive soil to provide improved conditions for infrastructure construction.

## 1. Introduction

Expansive soil is commonly known as “shrink-swell soil” and the construction of buildings on such soils are susceptible to differential settlement due to its high compressibility and weak shear resistance. The engineering properties of expansive soil such as liquid limit (LL), plasticity index (PI), compatibility, compaction, shear strength parameters (*C*, *Φ*), and consolidation coefficient have been successfully improved using chemical and mechanical stabilization techniques in a variety of applications. Chemical techniques primarily involve the addition of chemical binders such as cement [1,2,3], lime [4,5,6,7,8,9], fly ash (FA) [10,11,12,13,14], rice husk ash (RHA) [15,16,17], or polymers [18,19] to improve the quality and stability of clay soils. Mechanical techniques primarily consist of the application of reinforcement materials with compaction. Fibre types used for reinforcement include polypropylene, nylon, coir, palm, and plastic waste strips [20,21,22,23,24].

Indeed, waste materials have been increasingly applied around the world for soil stabilization or improvement because of the overproduction of wastes such as RHA, fly ash, plastics, and fibres, such as in India, where about 100 million tonnes of rice is produced annually, leading to a large quantity of RHA [25]. Using such waste material to improve soil has considerably reduced geotechnical problems [26,27]. Several studies have been conducted on the enhancement of soil behaviour using RHA (pozzolanic materials) in a specific ratio to improve the soil strength [28,29]. Roy Arian [15] investigated the effect of RHA in different percentages (10%, 15%, and 20%) on the properties of the soil such as compaction parameter (MDD) and (OMC), California bearing ratio (CBR) and Unconfined compressive stress (UCS). The results show that the RHA improved the CBR value and UCS of soil with 10% RHA content is recommended as optimum amount for practical purposes. Basha et al. [30] found that adding RHA to expansive soil reduced its PI and maximum dry density (MDD) and increased its optimum moisture content (OMC).

Kahn et al. [31] described the consolidation behaviour of clay slurries without additives and reported that the coefficient of consolidation and the coefficient of volume change decreased with increasing effective stress but increased with increasing void ratio and hydraulic conductivity. As the addition of RHA is likely to influence parameters such as the coefficient of consolidation and void ratio, the use of these materials could have a clear effect on the consolidation performance of expansive soil.

To date, few studies have focused specifically on consolidation performance used RHA as part of soil stabilizing techniques. The purpose of this study was to investigate the effect of using RHA as soil stabilizers to improve consolidation performance. To do so, the soil parameters were determined before and after adding different quantities of RHA (0%, 4%, 8%, 12%, and 16% by weight of soil). Based on the results, a relationship was derived to explain the effectiveness of RHA materials when used to improve expansive soil properties. The successful application of these materials not only leads to safe structural foundations that can be easily designed in expansive soil, but also represents a meaningful use of materials that are otherwise disposed of as waste.

## 2. Materials and Methods

### 2.1. Soil

The clay soil used in this study was collected from Ergun City, Inner Mongolia Province, North China. The soil samples were taken from a depth of 15 cm below the ground level and were kept in a plastic bag to retain their original water moisture content. The properties of the soil were then thoroughly studied in the laboratory of Northeast Forestry University (NEFU), China, according to prescribed standard methods, shown with their results in Table 1 [32,33,34,35,36,37]. The soil was classified as highly plastic clay (CH) according to the Standard Soil Classification system (USCS) and IS: 1498–1970 standards [38].

### 2.2. Rice Husk Ash

The RHA used in this study was from the Sanjiang Plain in Northeast China. The engineering properties of RHA are presented in Table 2, and the chemical properties of RHA are shown in Table 3. The RHA used in this investigation was passing throw the sieve No 200 (0.075 mm).

### 2.3. Sample Preparation

The clay soil was dried in an oven at 50 °C for 24 h then passed through a 0.425-mm (No. 40) sieve. The dried clay was then partitioned into 200-g samples to which three different quantities of distilled water were added. Thus, three samples with three different penetration depths of ≤5 mm, 9–11 mm, and 20 ± 0.2 mm were prepared. The samples were mixed by hand for three to five minutes and then placed into plastic bags that were stored in a curing container for 24 h. The RHA in quantities of 0%, 4%, 8%, 12%, and 16% by weight were later added and then used in the photoelectric liquid and plastic limit tests. In order to ensure that the triaxial and consolidation specimens were tested under similar conditions, each specimen was tested in the states of maximum dry density (MDD) and optimum moisture content (OMC). At least three samples were tested for each type of specimen and the average of the three resulting values was considered the final result.

### 2.4. Testing Methods

#### 2.4.1. Photoelectric Liquid and Plastic Limit Tests

**Figure 1:**
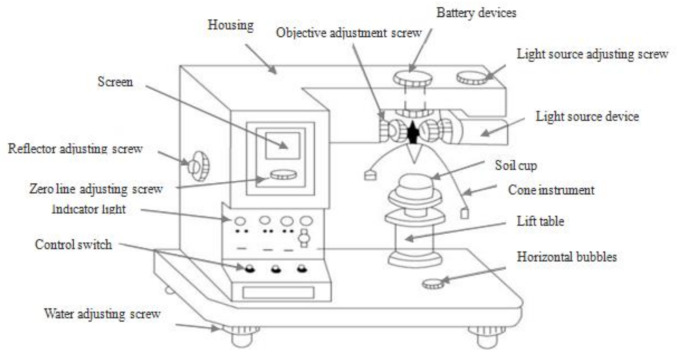
Schematic diagram of photoelectric liquid-plastic limit. “Influence of polypropylene fibre (PF) reinforcement on mechanical properties of clay soil” by M. M. M. Taha, C. P. Feng, and S. H. S. Ahmed, 2020. Advances in Polymer Technology, Copyright © 2020 Mazahir M. M. Taha et al. vol. 2020, 15 pages.

**Figure 2:**
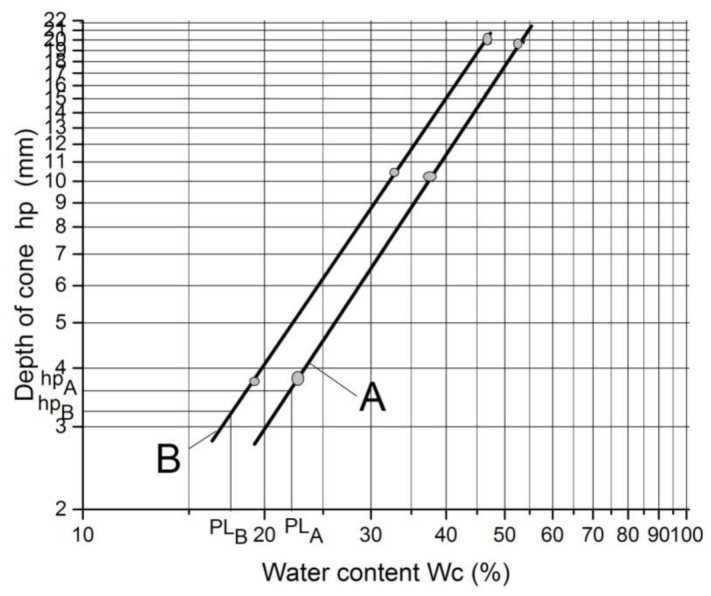
Relationship between the depth of cone (*h_p_*) and moisture content (*w_c_*).

The liquid and plastic limit tests were conducted using a GYS-2 photoelectric liquid-plastic tester from Nanjing Ningxi Soil Instrument Co., Ltd., in Nanjing, China. The liquid limit (LL) and plastic limit (PL) of the natural soil and RHA-reinforced soil samples were measured by the cone depth of the soil with the cone mass of 100 g and cone angle of 30 °C. Figure 1 depicts a schematic diagram of the photoelectric liquid and plastic limit testing apparatus used. The principle of the photoelectric testing method is based on the GB/T 50123-1999 standard soil test method and the JTG E40-2007 method of soil testing for highway engineering [39,40]. The sample was placed on the lifting table of the tester and the power was turned on so that the cone touched the specimen. The power to the electromagnet in the cone instrument was deactivated allowing the cone instrument to sink into the sample. The moisture content (*w_c_*) was measured corresponding to each cone depth (≤5 mm, 9–11 mm, and 20 ± 0.2 mm); thus, lines A and B in Figure 2 were plotted. The LL was determined from the test result as the moisture content at which the cone depth *h_p_* = 20 ± 0.2 mm. Using this LL, the cone depth *h_p_* was determined by Equation (1) [41]. The PL was then determined from Figure 2, based on the cone depth that was calculated from Equation (1) for each two straight lines A and B of a soil specimen.

*h_p_* = LL/(0.524 LL − 7.606) (1)

where *h_p_* is the cone penetration depth and LL is the liquid limit.

#### 2.4.2. Proctor Compaction Test

The soil samples were subjected to a Proctor compaction test according to ASTM D698-00a (2006) [36] to determine the MDD and OMC of the untreated and RHA-reinforced soils.

#### 2.4.3. Triaxial Test

Triaxial testing (CU) was performed using a TSZ-1 fully automatic triaxial test apparatus from Nanjing Ningxi Soil Instrument Co., Ltd., in Nanjing, China to determine the cohesion *C* and internal angle of friction *Φ* of the soil samples. The samples were prepared by static compaction at their respective optimum water content and maximum dry unit weight and formed in an 80 mm height with an inner diameter of 39.1 mm. The rate of axial strain was 0.800 mm/min, the axial force was 10 KN, and the pore pressures were 100, 200, and 300 kPa.

#### 2.4.4. Consolidation Test

The consolidation tests were performed using a fully automatic GZQ-1 testing apparatus from Nanjing Ningxi Soil Instrument Co., Ltd., Nanjing, China to determine the compressibility of the soil samples under different compressive stresses (12.5, 25, 50, 100, 200, 300, 400, and 800 kPa) and confining pressures. The complete equipment includes the consolidator, pneumatic controllers, multi-channel communication adapter, and data acquisition system. This system enabled standard and rapid consolidation testing to determine the void ratio (*e*), coefficient of consolidation (*Cv*), and the hydraulic conductivity (*k*). Samples for the consolidation tests were formed in a 20-mm deep ring with inner diameter of 61.8 mm.

#### 2.4.5. Microstructures Test

Scanning electron microscopy (SEM) technology combined with dispersive energy X-ray (EDX) is one of the best and most widely used methods to identify and characterize the composition of the microstructure and chemical characterization of the particles size. The SEM-EDX analysis was used to describe the microstructure changes in natural soil and soil treated with RHA (4%, 8%, 12%, and 16%) by weight of soil.

## 3. Results and Discussion

To understand the effect of RHA reinforcement on the properties of expansive soil, results of liquid and plastic limit tests, compaction tests, triaxial tests, and consolidation tests are discussed in this section.

### 3.1. Photoelectric Liquid and Plastic Limit Tests

**Figure 3:**
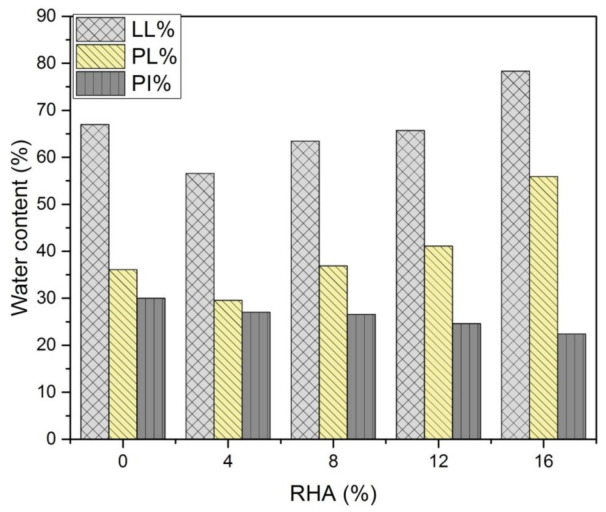
Effect of RHA on the water content of the soils.

The photoelectric liquid-plastic limit illustrated the LL, PL and PI of the untreated and treated soil in Figure 3. Figure 3 illustrate that the increased of RHA content, the LL and PL decreased and then increased while the PI decreased. The LL of the natural soil was 67%, which increased to 78% at 16% RHA (increased 16%). Similarly, the PL of the natural soil was 37%, which then increased to 56% at 16% RHA (increased 51%). The PI of the natural soil was 30% and decreased to 22% when treated with 16% RHA (decreased 25%). A similar behaviour when used fly ash with clay soil was reported by Jianqiao et al. [41]. The observed that the decreased of LL and PL when RHA are first introduced in the soil could be attributed to the reaction occurring between the soil and the R H A leads to a flocculation and agglomeration of clay particles. The increase in the size of the soil particles takes place due to the agglomeration of the clay particles and consequently the LL and PL decreased. In general, consistency limits significantly influenced by many factors, such as the properties of soils, specific surface area, and chemical reactions. Clearly, the addition of RHA improved the stability of the soil by reducing the PI.

### 3.2. Proctor Compaction

**Figure 4:**
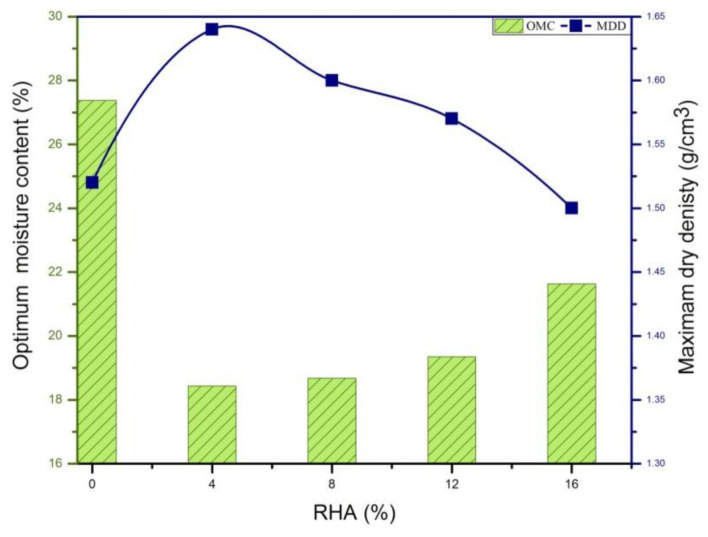
Dry density and optimum moisture content for soil and RHA.

The proctor compaction test results of the natural soil and treated soils are shown in terms of OMC and MDD in Figure 4. Figure 4 indicates that the OMC decreased with the increased in RHA. Comparing the natural soil and treated soils, the OMC of the natural soil was 27% and decreased and then increased to 22% with 16% RHA, and the MDD increased and then decreased with increased in RHA: the MDD of the natural soil was 1.52 g/cm^3^ and decreased to 1.49 g/cm^3^ at 16% RHA. This decrease can be attributed to the low specific gravity of RHA: the specific gravity of the natural clay soil particles was 2.58, and these were replaced by RHA particles with a lower specific gravity of 1.66. Similar behaviour has been reported by several other researchers [30,42].

### 3.3. Triaxial Compressive Testing

**Figure 5:**
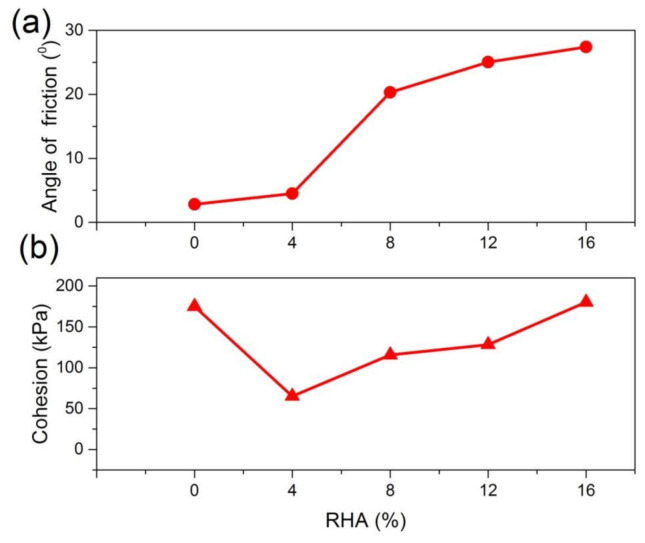
The relationship between shear strength parameters of soil and RHA: (**a**) angle of internal friction; (**b**) cohesion.

Figure 5 presents the shear parameters of the specimens with different RHA contents. Figure 5a shows that increasing RHA increased the maximum internal angle of friction (*Φ*) of the natural soil from 2.83° up to 26° with 16% RHA. A reasonable explanation for the increased observed in the angle of internal friction could be attributed to the increase in the size of the soil particles takes place due to the agglomeration of the clay particles when the soil particles interaction with RHA. Nabanita et al. [43] demonstrated that the angle of internal friction increased with increase in RHA content. Figure 5b indicates that the cohesion (*C*) of the soil decreased when RHA are first introduced in the soil this could be attributed to replacement of soil by the RHA and also to RHA reacted with soil which agglomerates the particles into a larger one. The maximum cohesion (*C*) of treated soil was 180.25 kPa obtained with the optimal RHA content of 16%. These results are similar to those reported in previous study [43,44].

### 3.4. Consolidation

**Figure 6:**
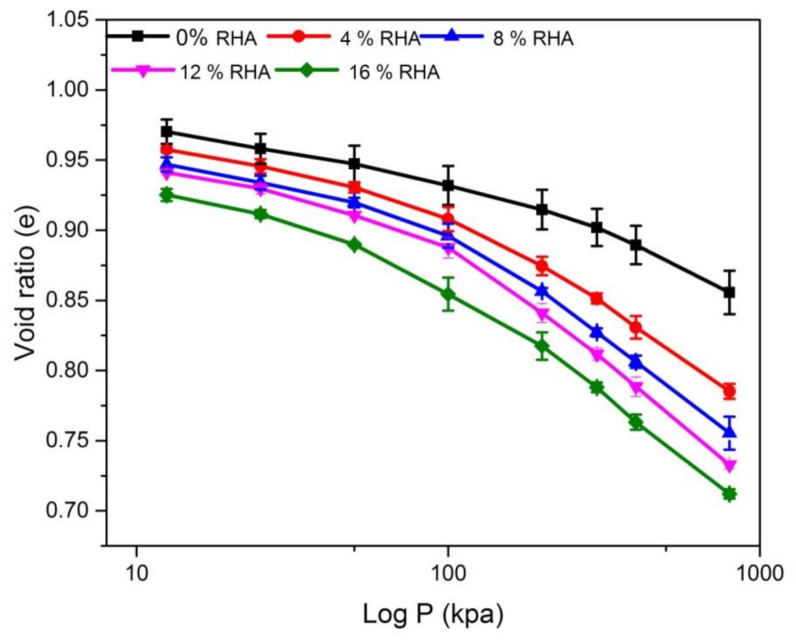
Void ratio and log P pressure for different percentage of RHA.

Zeng et al. [45] presented an important concept that compressibility, shear resistance and hydraulic conductivity are related to each other and represent the same physical phenomenon. Accordingly, a consolidation test was conducted using the oedometer provided data to analyse the variations of stress applied during the compression process with respect to compressibility and hydraulic conductivity. Previous studies on the compressibility of clay serve as a reference to evaluate the void ratio (e) of clay soil. To study the potential for consolidation, the void ratio (e) of untreated and treated soil was found as a function of effective pressure (log P, e), the results are represented in Figure 6. Figure 6 shows the void ratio with standard deviation of the treated and untreated soil. The void ratio and the compression curves of the treated soil plotted below the untreated soil curve: the void ratio decreased varying from 0.96 to 0.86 for natural soil and varying from 0.93 to 0.72 for treated soil with 16% RHA. The observed decrease in void ratio could be attributed to the RHA particles filled the pores and reduced the total porosity of the soil. Phanikumar and Singla [21] have shown that the equilibrium void ratio of natural soil is the highest, while the treated expansive soils have lower equilibrium void ratio when saturated after being submerged. Similar behaviour of clay slurries has been reported by Khan et al. [31].

**Figure 7:**
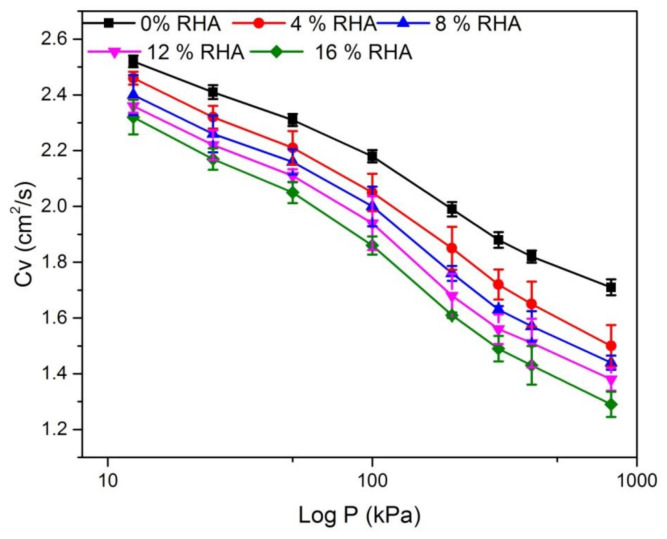
Coefficient of consolidation (*C_V_*) relation with different percentage of RHA.

The relationships of log P consolidation pressure and the coefficient of consolidation (*C_V_*) for the soils treated with RHA are presented in Figure 7. The *C_V_* indicates the rate of consolidation of expansive soil is calculated using the void ratio from the compression curves in Figure 6. Figure 7 shows the coefficient of consolidation with standard deviation of the treated and untreated soil. Figure 7 illustrates that the *C_V_* decreased from a range between 2.52 cm^2^/s and 1.71 cm^2^/s for the natural soil to a range between 2.33 cm^2^/s and 1.30 cm^2^/s for 16% RHA. The observed decrease in *C_V_* is likely due to RHA causing tangles and frictional effects within the soil structure, and also due to the initial conditions of expansive soil (such as water content or void ratio), formation of a torsional flow path in the expansive soil, or the expansive soil intrinsic properties (such as specific gravity, particle size distribution, or mineral composition). Khan and Azam [30] also assert that with increased of effective stress, the *C_V_* behavior of the clay slurries decreased, while increased with the increased of (e) value.

**Figure 8:**
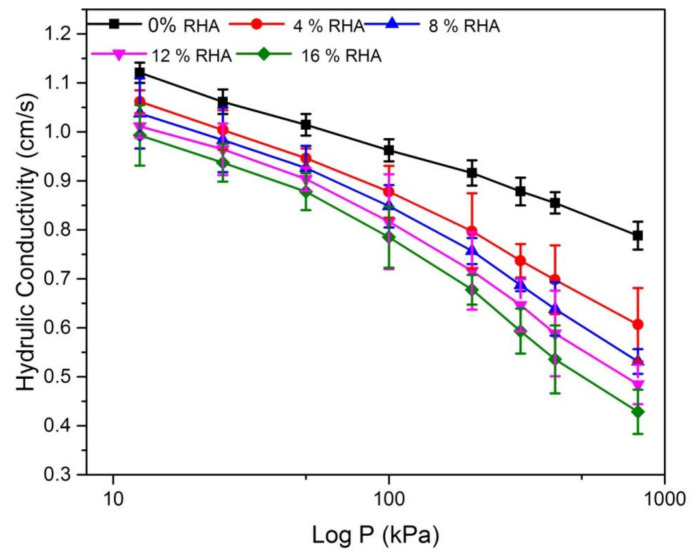
Hydraulic conductivity (*K*) relation with different percentage of RHA.

Similar to the *e*–log *P* pressure curve, it can be seen from the *k*–*e* curve shown in Figure 8. Figure 8 shows the hydraulic conductivity with standard deviation for the treated and untreated soil. It shows that the hydraulic conductivity (K) of natural soil specimen is higher than that of the treated soils. With the increased void ratio the hydraulic conductivity increased and decreased with the increased of RHA content, which corresponds to the contrast trend observed above for void ratio and consolidation coefficient. Within the study, the hydraulic conductivity of the natural soil ranged from 1.12 cm/s to 0.83 cm/s and the hydraulic conductivity of the treated soil ranged from 0.99 cm/s to 0.43 cm/s. As mentioned earlier, this decrease in hydraulic conductivity could be attributed to the RHA particles filled the pores and reduced the total porosity of the soil. Additionally, the scattering of soil particles at high void ratios contributed to more potential pockets of air in the sample, increasing hydraulic conductivity. These results are consistent with some previous studies [45,46].

### 3.5. Microstructure Analysis

**Figure 9:**
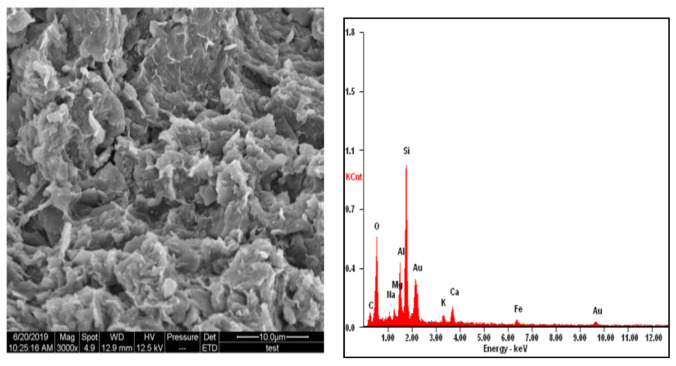
SEM and EDX diagram of the natural soil.

**Figure 10:**
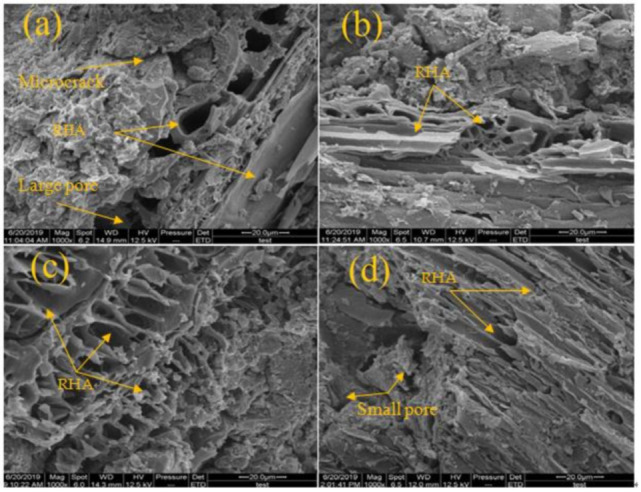
SEM micrographs (**a**) RHA 4% (**b**) RHA 8% (**c**) RHA 12% (**d**) RHA 16%.

**Figure 11:**
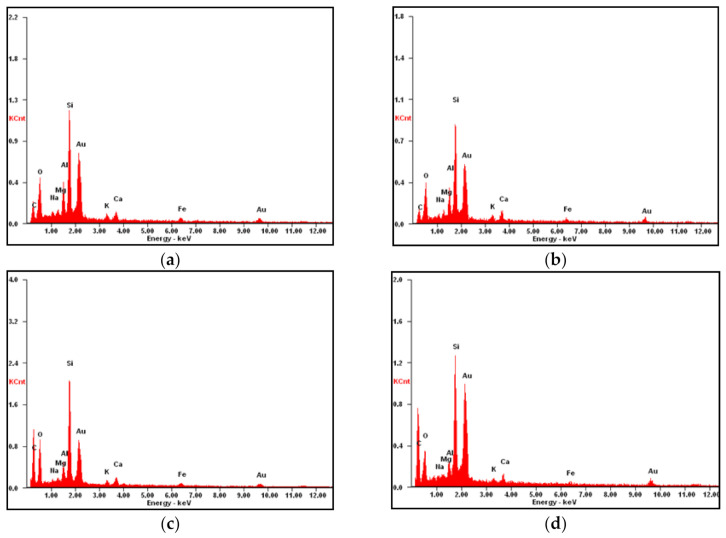
EDX diagram of treated soil with RHA (**a**) RHA 4%, (**b**) RHA 8%, (**c**) RHA 12%, (**d**) RHA 16%.

The results of scanning electron microscope (SEM) and energy dispersive X-ray (EDX) was shown in Figure 9, Figure 10 and Figure 11. Figure 9 shows the SEM and EDX images of the natural soil. Figure 10 shows the SEM images of RHA. The images show that the microstructure of treated soil samples changes due to the stabilization process and the reaction between the soils and RHA content. Figure 10a shows that at the interface of the soil and RHA specimen, a large pore has been seen. The large pore and microcracks on the surface of soil samples and the RHA reacted with soil and agglomerates the particles into a larger one, which caused pockets. This could be responsible for the reduction in the cohesion and increased the angle of internal friction in Section 3.3.

EDX analysis was performed on different RHA particles with distinct morphologies and the results are shown in Figure 11 and Table 4. EDX data indicate that RHA particles contained varying amounts of elements i.e., O, C, Na, Mg, Al, Si, K, Ca, and Fe. Figure 11a show high values of Ca, Al, and O and low value of C. The variation in these elements may be due to replacement of soil particles by RHA. This could be responsible for the reduction in the cohesion when RHA are first introduced in the soil in in Section 3.3.

## 4. Conclusions

A series of laboratory tests were performed to evaluate the effectiveness of adding RHA to improve the properties of expansive soil in terms of compaction property, shear parameters, and consolidation performance. From the results of these tests, the following conclusions can be drawn:

- The PI decreased with the increase in RHA content. This improvement in the PI is due to an increase in strength and a reduction in compressibility.
- Adding RHA decreased the MDD of the clay soil specimens from 1.52 g/cm^3^ for the natural soil to 1.49 g/cm^3^ for soil treated with 16% RHA. The OMC decreased from 27.38% for natural soil to 21.63% for soil treated with 16% RHA.
- In terms of shear parameters, the friction angle *Φ* of the clay soil specimens increased considerably when adding RHA, whereas the cohesion *C* decreased overall with increasing of RHA.
- The void ratio for clay soil decreased with adding RHA, the natural clay soil specimens void ratio *e* = 0.96 and the specimens treated with 16% RHA void ratios *e* = 0.93.
- The determined relationship between the consolidation coefficient *C_v_* and the consolidation pressure was similar for the natural soil as well as the RHA treated soils, the value of *C_V_* decreased from 2.33 cm^2^/s to 1.30 cm^2^/s for 16% RHA.
- The hydraulic conductivity *k* decreased with increasing RHA. The *k* value for the natural soil between 1.12 cm/s and 0.83 cm/s decreased to a value between 0.99 cm/s and 0.43 cm/s for 16% RHA.
- From the results of this study, it was determined that the use of RHA improved the properties and consolidation performance of expansive soils as the porosity and density of the RHA were lower than the natural soil.

## Figures and Tables

**Table 1:** Index Properties of soil.

Property	Value	Standard Name
Specific gravity	2.58	ASTM D854
Moisture content (%)	13.35	ASTM D2216
**Grain size analysis/Hydrometer**		ASTM D422
Gravel (%)	1.11	ASTM D422
Sand (%)	9.89	ASTM D422
Clay (%)	89	ASTM D422
Liquid limit (%)	67	ASTM D4318
Plastic limit (%)	36.09	ASTM D4318
Plasticity index (%)	30.91	ASTM D4318
USUC Classification	CH	ASTM D2487
**Compaction Parameters**		ASTM D698
Optimum moisture content (%)	27.38	ASTM D698
Maximum dry density (g/cm^3^)	1.52	ASTM D698
**Shear Strength Parameters**		ASTM D2850
**C** (kN/m^2^)	175	ASTM D2850
*Φ* (°)	2.83	ASTM D2850

**Table 2:** Engineering properties of RHA.

Properties	RHA
Colour	Dark Gray
Specific gravity	1.66
Plasticity index (%)	Non plastic
Loss on Ignition (%)	1

**Table 3:** Chemical properties of RHA.

Oxide Compounds	RHA (%)
Calcium oxide (CaO)	3
Silica (SiO_2_)	21.4
Alumina (Al_2_O_3_)	19.8
Iron oxide (Fe_2_O_3_)	0.6
Magnesia (MgO)	1.5
Sodium oxide (Na_2_O)	1.59
Potassium oxide (K_2_O)	1.13
Sulfur trioxide (SO_3_)	0.84

**Table 4:** Concentration statistics of element in surface of the soil.

Element	RHA (4%)	RHA (8%)	RHA (12%)	RHA (16%)
C	24.88	32.13	47.99	55.30
O	26.35	22.50	22.51	16.50
Na	01.65	00.61	00.48	00.45
Mg	01.40	00.80	00.59	00.37
Al	06.52	03.22	03.04	02.00
Si	25.19	16.57	17.90	18.34
K	02.62	01.80	01.39	01.19
Ca	04.18	03.80	02.74	02.53
Fe	07.19	04.77	03.36	03.32

## Data Availability

Not applicable.
